# Professional Accountability of Caring for Patients with COVID-19: A Phenomenographic Study

**DOI:** 10.3390/healthcare11162269

**Published:** 2023-08-11

**Authors:** Li-Chin Chen, Shu-Ling Yeh, Hui-Ling Lee, Chun-Chih Lin, Suzanne Goopy, Chin-Yen Han

**Affiliations:** 1 Department of Nursing, New Taipei Municipal TuCheng Hospital, Chang Gung Medical Foundation, New Taipei City 236, Taiwan; judy5612@cgmh.org.tw (L.-C.C.); cclin01@mail.cgust.edu.tw (C.-C.L.); 2 Department of Nursing, Chang Gung University of Science and Technology, Taoyuan 333, Taiwan; lhling@mail.cgust.edu.tw; 3 Department of Nursing, Taipei Medical University, Taipei 110, Taiwan; 4 Department of Nursing, Chang Gung Memorial Hospital at Keelung, Keelung 20401, Taiwan; q22122@cgmh.org.tw; 5 Usher Institute, University of Edinburgh, Edinburgh EH8 9AG, UK; sgoopy@ed.ac.uk

**Keywords:** professional accountability, nurses experience, COVID-19, phenomenography

## Abstract

The purpose of this study was to explore nurses’ care experiences for COVID-19 patients during the pandemic in Taiwan. The qualitative approach of phenomenography was used. Thirty-four nurses were recruited from two assigned hospitals in which COVID-19 patients were treated in Taiwan from July to May 2021. The method of data collection in the study involved a semi-structured interview and drawing. Interviews were audio-recorded and transcribed verbatim. Phenomenographic analysis was used to analyze the qualitative data. Four categories of description of experiences of caring for COVID-19 patients were identified: facing uncountable stresses from all sides, strict implementation of infection control interventions to provide safe care, confronting ethical dilemmas and making difficult decisions, and reflecting on the meaning of care in nursing. Professional accountability was the core theme found to represent the central meaning of nurses caring for COVID-19 patients. Nurses were under enormous stress while caring for COVID-19 patients during the pandemic and were negatively affected physically, psychologically, and socially. Professional accountability in caring for COVID-19 patients can be enhanced through adequate support from nursing managers and by in-service training designed to update knowledge and skills related to infection control intervention.

## 1. Introduction

An infectious disease outbreak can develop into a large-scale public health crisis that threatens human life [1]. On 30 January 2020, COVID-19 was declared an international public health emergency by the World Health Organization (WHO) [2], as rapidly increasing numbers of patients diagnosed with COVID-19 impacted healthcare systems and nurses’ physical, psychological, and psychosocial health worldwide [3,4,5]. Nurses comprise the largest group of healthcare providers and are also those in close contact with infected patients. As providers of first-line patient care, nurses have been particularly affected, both physically and mentally, by their work with COVID-19 patients [6,7]. It is therefore important to explore their experiences during the COVID-19 pandemic.

The effects of the COVID-19 outbreak have been described as unprecedented [4,8,9]. Nurses were on the front line of the response to the health crisis, facing a high risk of infection [1,6]. The prevalence of COVID-19 infection among nurses is 48%, which is the highest rate among healthcare providers [10], but treating patients with COVID-19 caused various other problems, such as high levels of stress, fear, mental burnout, and social isolation [11,12,13,14,15,16,17,18,19]. Nurses also suffered physically, as evidenced by the high infection rates in the profession [1,6]. International studies have shown that nurses have been particularly affected by the experience of working with patients with COVID-19 [6,7,20]. Taiwan’s studies demonstrated that the effects of the COVID-19 pandemic on nurses and healthcare workers included fear of the uncontrollable pandemic, and upon being infected, a moderate level of stress, strictly limited social activities, and being discriminated against by their family members [21,22]. To be better prepared for future outbreaks, it is crucial to develop awareness of the experiences of nurses caring for patients with COVID-19.

By 12 July 2021, the number of confirmed cases of COVID-19 in Taiwan had reached 15,500. Compared with the more than 1.86 billion people infected with the coronavirus and more than 4 million deaths worldwide, the situation in Taiwan was relatively stable and well controlled during the COVID-19 pandemic period. This might account for the minimal attention paid to nurses’ experiences in the outbreak in Taiwan. From clinical observations, nurses and hospitals in Taiwan were under-prepared during the early stages for an outbreak of a novel infectious disease, including an identified lack of adequate knowledge and professional skills to deal with the disease and difficulty in obtaining accurate information, as well as an insufficient number of beds, inadequate staffing and equipment, unacceptable workloads, and poor routine and special staff training. While the impact of COVID-19 on nurses has been studied extensively [4,13,15,16,17], limited studies have investigated nurses’ experiences from different methodological perspectives. Most published qualitative studies applied interviews to gain insights into the nurses’ experiences. This study combined drawings and interviews presented by nurses who faced the COVID-19 pandemic and verified nurses’ dedication to caring for patients with infectious diseases in the context of a public health crisis. The results of this study can serve as the basis for future planning to assist healthcare providers in the management of infectious disease outbreaks.

## 2. Materials and Methods

### 2.1. Rationale and Aims

The purpose of this study was to investigate nurses’ experiences of caring for patients with COVID-19 in Taiwan. This is a phenomenographic study design. Phenomenography was used to gain insights into nurses’ interpretations of caring for patients with COVID-19 during the pandemic. Phenomenography is a research methodology that aims to describe, analyze, and understand experiences [23]. It identifies reality with phenomena that are present in experience and seeks to describe the major features of the various ways in which people experience a phenomenon [24]. The results of a phenomenographic study are categories of description and an outcome space. Categories of description represent the central meanings of a phenomenon. They describe similarities and differences in meaning and reflect the qualitatively different ways in which phenomena are understood [24,25]. The outcome space is a diagrammatic representation of the logical relationships between the categories of description and the investigated phenomenon [26]. In this study, phenomenography facilitated the development of a qualitative map of the ways in which nurses experienced and understood caring for patients with COVID-19.

### 2.2. Recruitment, Criteria, and Sampling

This study was conducted at two hospitals in Taiwan—one private and one public. The private hospital is a medical center with 3000 beds, while the public hospital, also a medical center, has a capacity of 1100 beds. Both hospitals were assigned by the Taiwan CDC to treat all levels of severity of the patients with confirmed COVID-19 from the beginning of the outbreak. Purposive sampling was used to select participants for the study to ensure that they were willing to fully describe their experiences with the phenomenon. The eligibility criteria for inclusion in the study were as follows: (1) registered nurses aged 20 years or older; (2) who were providing primary care to patients with COVID-19 at a negative pressure unit; and (3) whose primary language was Mandarin. According to the Taiwan CDC policy, patients with confirmed COVID-19 visited the emergency department and then were admitted to the negative pressure unit for further treatment. A total of 34 participants were recruited from three units of the two participating hospitals. All nurses who worked at the negative pressure units received an email with a written document that explained the study. The potential participants made contact with one of the researchers (C.Y.H.) via email or phone. The researcher fully explained the purpose and procedures of the study to potential participants. Convenient interview times for the participants then were arranged.

Two methods of collecting qualitative data were employed: interviewing and drawing, in the present study. Semi-structured interviews are commonly used to collect data in phenomenographic studies [24,27], especially to obtain descriptions of the participants’ experiences in their own words. The aim of the interview in the present study was to seek variations in participants’ experiences and their understanding of the phenomenon under investigation. The use of interviews allowed the participants to provide rich descriptions of their experiences, which can be used to validate the researcher’s interpretation of the theme of the study [24]. Therefore, the same researcher (C.Y.H.) conducted all interviews at two participating hospitals in the study, which not only provided rich descriptions of nurses’ experience in caring for patients with COVID-19, but also addressed the reliability of the study.

Additionally, each participant was asked to complete a short questionnaire with information on age, gender, educational level, and years of nursing experience before being interviewed. The interviews began with a straightforward question (such as “What was work like today?”) to encourage the participants to start reflecting on their practice. The participants were then asked to focus on their practice related to caring for patients with COVID-19. Questions included “Did you care for any patients with COVID-19 today?” and “Could you provide some examples of how you care for patients with COVID-19?” The aim was to elicit the participants’ views on the issues in question [28]. The interview guide is attached as an Appendix A. Each interview lasted between 40 and 65 min. All interviews were conducted in a secured room that ensured privacy and provided a safe and stress-free environment in which the participants could talk freely about their experiences of caring for patients with COVID-19. Interviews were recorded and transcribed verbatim by the researcher who conducted them.

Drawing is another method of collecting data in phenomenographic studies [24]. Drawing a picture stimulates various responses that encourage further examination of the phenomenon from the participants’ perspective and their experience. Drawing also demonstrates various responses that encourage further examination of an individual’s conceptualization and experience of a phenomenon [29]. Drawing pictures can gain prominence in qualitative research and present how the people see the phenomenon under the investigation [24,29]. Therefore, drawing adds rich data for phenomenographic studies. At the end of each interview, each participant was asked to draw a picture related to caring for patients with COVID-19 and then explain what they were trying to express. Where necessary, the interviewer asked for clarification. Only four participants completed their pictures. The reasons participants gave for not completing the drawing included feeling incapable of drawing, and some simply refused to try. In such cases, the researcher gave the participant paper to draw a picture at home, but unfortunately, no one returned a picture. The data were collected between July 2020 and May 2021, the period from the beginning to the first wave of the COVID-19 outbreak in Taiwan. Data collected from the participants during this period shaped their experiences of caring for patients with COVID-19.

### 2.3. Data Analysis

The data were analyzed in seven steps used in phenomenography: familiarization, condensation, comparison, grouping, articulating, labeling, and contrasting [27]. To familiarize themselves with the content, the researchers first read the interview data several times. The condensation step involved the identification of significant statements related to the participants’ understanding of the phenomenon in question. In the comparison stage, the meaning units of the interview data were compared to identify similarities and differences. The grouping stage started when similarities and differences were identified. The researchers identified a logically related set of four categories of description, and the outcome space began to emerge. The articulating stage involved initial attempts to explain the participants’ experiences of the categories of description. The researchers repeated this process several times until the categories of description reflected the participants’ understanding and experiences. In the labeling stage, the researchers tentatively identified and named categories of description. Finally, in the contrasting stage, similarities and differences between these categories of description were identified to determine the logical relationships between them and the investigated phenomenon. The researchers revisited the interview data in the categories of description many times until a diagrammatic representation of the phenomenon was established.

The aim of the phenomenographic data analysis was to describe the phenomenon in terms of participants’ essential meanings and awareness while considering the overall context, meaning, and perspective. Both the verbal and non-verbal data are important for phenomenographic analysis [24]. The interview transcripts were coded manually by two researchers separately, who then met and compared the codes and categories emerging from the data. For any differences, the consensus was reached through discussion. While coding was conducted separately, the interview data were discussed throughout the process. Upon completion of 30 interviews, no new categories emerged from the data. To confirm this, an additional four interviews were conducted. The sample size of 34 participants was found sufficient to generate rich data and reach saturation.

The rigor of the study was ensured through faithful description, interpretative awareness, and communicative validity [30]. Throughout the research process, the researchers represented the participants’ intentional relationships to their experiences in a way that was as faithful as possible to their conceptions of reality [28], During the interviews, the researcher put aside her own experience of the phenomenon, encouraging the participants to describe their experiences in their own words and treating all experiences as being of equal value. Communication validity was attained by generating data through in-depth and focused dialogue [30].

### 2.4. Ethical Considerations

This study was approved (IRB number 202000525B0) by the institutional review boards of the hospitals from which the participants were recruited. Participation was voluntary, and the participants were given assurances of confidentiality, anonymity, and the right to withdraw at any time with no consequences for their ongoing employment. All participants provided written informed consent.

## 3. Results

### 3.1. Characteristics of Participants

A total of 34 nurses participated, including 32 females and 2 males. The mean age was 30.0 ± 4.83 years (range from 22 to 42 years). In total, 28 participants had a bachelor’s degree. A total of eight were working in the medical ward, eight in the intensive care units, and eight in the emergency department of the hospital. The number of years in nursing experience ranged from 1 to 21 years with a mean of 7.61 ± 4.60 years (Table 1). The thirty-four nurses interviewed are identified as RN1 to RN34, rather than by name, in order to maintain confidentiality and anonymity.

### 3.2. Categories of Desctiption

Phenomenographic analysis of the data identified four categories of description for nurses’ experiences of caring for patients with COVID-19: facing stresses from all sides; implementing strict infection control interventions to provide safe care; confronting ethical dilemmas and making difficult decisions; and reflecting on the meaning of care in nursing. Each of these categories of description is elaborated below, and the relationship between the categories is presented in the outcome space.

#### 3.2.1. Categories of Description One: Caring for Patients with COVID-19 as Facing Stresses from All Sides

In the early phases of the COVID-19 outbreak, nurses faced the stress of caring for COVID-19 patients and the uncertainties of working with an unfamiliar disease under different work conditions and protocols. Nurses were concerned about the unknown aspects of the disease, as shown in the following comments made by nurses working in outdoor fever-screening stalls and negative pressure isolation units:

*RN5: I was extremely scared. I’ve never met a disease like this before, and I’ve scrubbed my hands to the point that they crack… and was soaked in sweat from head to toe after the shift without realizing this happened.*

*P12: …made sure I’d put on the protective clothing correctly at work. In the summer, I was soaked in sweat from head to toes after my shift. Perhaps because I was so scared about contracting the disease, I didn’t realize I was all wet.*

*RN18: I had nightmares for days when I first started caring for them, and I’ve had dreams about getting infected myself.*

In addition to the stress of facing the novel infectious disease at work, nurses were also distressed by the possibility of contracting the disease or spreading it to family members. Nurses experienced worry and social pressure while working with COVID-19 patients, as exemplified by the following comments:

*RN4: I heard from my mother that the neighbors stay away (from my family) like they are avoiding the plague. All I could do was console my parents. I feel so guilty about the potential COVID-19 exposure risk to my family.*

*RN7: For some time, I’ve intentionally made up reasons for not going home because I was worried about the consequences of bringing the virus into my home and infecting my kids.*

*RN16: I had nightmares for days when I first started caring for them, and I’ve had dreams about getting infected myself (smiling wryly).*

#### 3.2.2. Categories of Description Two: Caring for Patients with COVID-19 as Strict Infection Control to Provide Safe Care

Nurses indicated that during the early phases of the outbreak, hospitals promptly provided relevant information and skills training for COVID-19 patient care. Colleagues practiced the skills among themselves. During patient care, they reminded each other about the infection control protocols and provided mutual assistance. The actions of looking out for one another and ensuring that all measures were strictly enforced not only accelerated patient recovery but also reduced the risk of exposure among of health professionals.

*RN15: …hospitals quickly offered online lessons about infection control strategy. No one liked online learning, but we all participated and discussed the lessons with each other whenever there was a problem. We just wanted to practice well infection control intervention in the care process, as this ensured not only the safety of patients but everyone else.*

*RN6: I was assigned to work here (in the negative pressure isolation unit) from the surgical ward. I saw that everyone had put on the PPE (personal protective equipment) properly without any complaints, even when our hands cracked or we had deep marks on our faces made by N95 masks. This was done so that the patients and colleagues were not exposed to the risk of being infected.*

**Figure 1:**
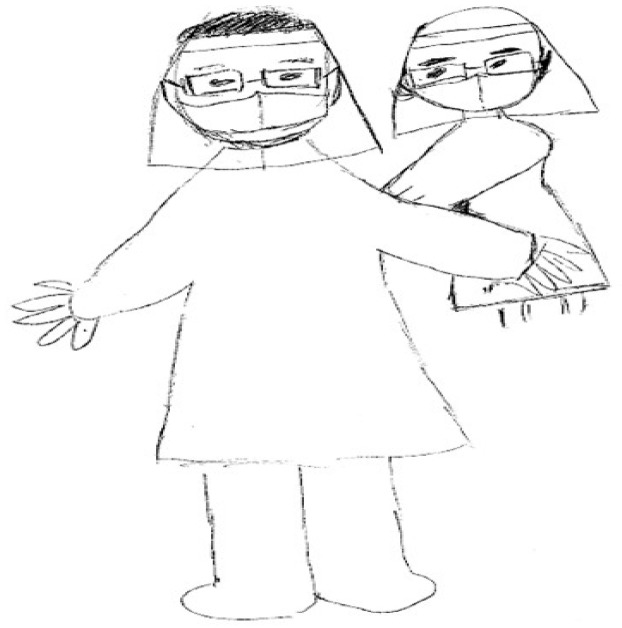
Strict implementation of infection control measures to provide safe care.

In Figure 1, the nurse (RN22) depicted colleagues helping each other to put on personal protective equipment and preparing the care for patients with COVID-19 at the beginning of the shift. She explained that the correct use of personal protective equipment is important for patient safety and that careful checking of each other’s personal protective equipment can prevent a breach in safety procedures. The nurse emphasized how they ensure the implementation of the infection control procedure while wearing the personal protective equipment. She further explained that colleagues in her units commonly remind each other the best practice of infection control procedure during the shift.

#### 3.2.3. Categories of Description Three: Caring for Patients with COVID-19 as Confronting Ethical Dilemmas and Making Difficult Decisions

Many regulations must be followed when caring for patients with COVID-19. Nurses are placed in the difficult situation of trying to make reasonable, ethical, and lawful decisions, especially when patients are close to the end of their lives. Participants were overwhelmed emotionally when making decisions in such situations, as described in the following excerpts:

*RN4: I’ve come across two beds (patients) that needed assistance at the same time, and I had to decide which one to go to first. They were really difficult decisions to make. I had to “gear up” and get into the negative pressure room within such a short time.*

*RN15: It stay imprinted in my mind because she was the index patient and transmitted the disease to several of her family members. At the final moment when she was in critical condition, her family was forbidden by law from being present and said their final goodbyes via the screen. And then, her body had to be cremated within the mandatory time.*

*RN21: I’ve never seen patients deteriorate so quickly in such a short time… and I have to immediately decide what I need to do. When their family members were also in isolation, crying over the phone or through the screen and asking us what to do, we were also grieving internally despite responding to them calmly. They couldn’t be with their dying loved ones.*

**Figure 2:**
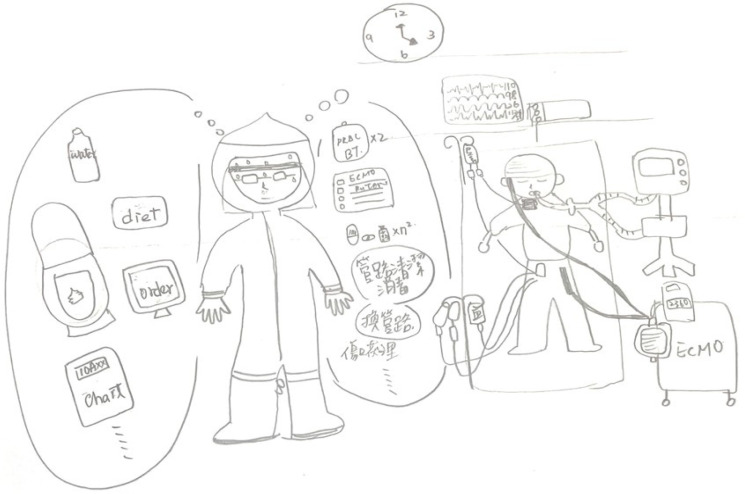
Confronting ethical dilemmas and making difficult decisions.

In Figure 2, the nurse (RN3) depicted the unstable condition of a patient with COVID-19 in an ICU. The shift had ended (at 4 PM), but many important things still needed to be done to save the patient’s life, such as new orders, a blood transfusion, wound dressing and draining care (in Chinese). The patient’s vital signs were getting worse, but the nurse found it difficult to prioritize and could not go any faster due to the restrictive full-body personal protective equipment, which made her sweat. The nurse described that the situation not only happened before the shift ended, but it was also a daily routine to face ethical issues when caring for the patients in the negative pressure unit during the COVID-19 pandemic.

#### 3.2.4. Categories of Description Four: Caring for Patients with COVID-19 as Reflecting on the Meaning of Nursing

The nurses stated that the main focus of caring for patients with COVID-19 was to meet their needs throughout their hospital stay, starting from the moment that COVID-19 infection was suspected to a definitive diagnosis being given. The modalities of caring for patients in the negative pressure isolation room differed starkly from those in standard wards. Family members traditionally play an important role as co-caretakers during hospitalization in Taiwan. During the present pandemic, nurses have had to provide all the COVID-19 patient care in the negative pressure unit without family involvement. The experiences of provision and engagement in caring for COVID-19 patients are different from the previous nursing experiences. These changes led the nurses to rethink the meaning and the role of nursing, as described by the following participants:

*RN17: An older (sick) man waited while I put on PPE (personal protective equipment) before going to the isolation room. He only wanted to use the bathroom and needed help. He was in tears and said “thank you” many times… he told me he didn’t want to soil the bed sheet. I was so moved and understood the meaning of caring.*

*RN11: …always made sure that everything was well prepared before going into the negative pressure room so that I could tend to all the patient needed. It takes a long time putting on the PPE and I couldn’t keep the patient in pain, waiting for so long… I was the only person who could help (him).*

### 3.3. The Outcome Space: Professional Accountability in Nursing

**Figure 3:**
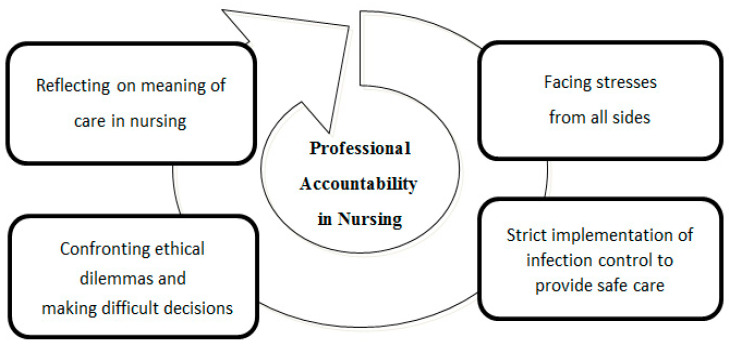
Professional accountability as the central meaning of care for patients with COVID-19.

Professional accountability in nursing is understood as the obligation to promote the welfare and wellbeing of the patient through nursing best practice. Participants were aware that caring for patients with COVID-19 was not only part of their duty of care, they were also accountable for their actions and for providing exemplary quality of care. Despite the multiplicity of stresses they were experiencing during the pandemic, they actively participated in online learning about infection control strategies and implemented the relevant interventions to ensure safe care. The experience of participating in difficult decision making while implementing different care models provided an opportunity for nurses to rethink the meaning of being a professional nurse. Professional accountability emerged as the core theme in nurses’ accounts of their experiences of the care process. Faced with stress from all sides and having to deal with ethically challenging situations, they found support in following the guidelines in care models and practicing holistic nursing care with patients with COVID-19 (Figure 3).

## 4. Discussion

In the present study, nurses reported that they faced multiple stresses while caring for patients with COVID-19. This result is consistent with the findings from other studies, Nurses are under immense physical, psychological, and social stress while caring for patients with infectious disease [15,31,32]. In Turkey, nearly 50% of emergency nurses reported experiencing above-average levels of stress during the COVID-19 pandemic [6]. Healthcare professionals in the UK also experienced despair, helplessness, anxiety, and depression [7,8,9,10,11,12]. In Spain, they were overwhelmed by exhaustion and negative emotions [20]. In a Brazilian study, five-hundred and seventy-two nursing professionals responded, showing that 26.4% presented a sleep disorder and 17.3% presented burnout [14]. A study of 1257 health professionals caring for patients with COVID-19 in China found that they suffered from a variety of symptoms such as depression, anxiety, and sleeplessness [15]. Other studies have documented healthcare professionals’ concerns about contracting the disease and passing it on to their families [31,32,33,34]. Compared with an earlier study related to the experiences of caring for patients in a negative pressure isolation ward, the study has demonstrated that nurses are indeed at increased risk of infection due to contact with patients and of transmitting the disease to their family members [35]. In addition to these concerns, other research indicates that health professionals are also worried that they and their family members might be more likely to be placed in mandatory quarantine than other workers, which raises the issue of potential discrimination due to their profession [33,34,36]. These aforementioned worries and concerns are the same as those in the results of the present study.

The significance of implementing infection control strategies that provide safe care for COVID-19 patients is critical, as mentioned by participants in the present study. Investigating nurses’ challenges during the infectious disease outbreak identified a lack of adequate knowledge and skills and an inability to obtain accurate information as common problems [37]. The control of infectious disease outbreaks is critically dependent on the risk management capabilities of healthcare providers [15,38]. In line with this study, previous research has also identified insufficient knowledge and skills and difficulty obtaining information as common problems facing nurses during the pandemic [39,40]. For example, 35% of front-line health workers in the UK expressed a need for support and felt that their demands were not being met [7]. In-service training programs are known to be effective for increasing the awareness and accuracy of the application of infection control intervention. Such training helps nurses provide safe care to patients and avoid the risks associated with working in a danger zone [35]. The nurses in the present study emphasized the importance of strict implementation of infection control strategies. This finding reflects the actions of the Taiwanese government, which was aware of the potential problems associated with caring for patients with COVID-19 by early 2020. They responded by ensuring that hospitals promptly provided nurses with online in-service training on infection control and adequate supplies of personal protective equipment.

Nurses in this study described the ethical dilemmas and difficult decisions involved in the care process. The literature documents ethical dilemmas for nurses that have arisen during the pandemic, including the contradiction between responsibility and reciprocity. The greater awareness of these ethical issues is needed [4,40,41]. During the SARS pandemic in Taiwan, nurses reported having to make difficult decisions related to patient care; they were subject to the ethical requirement to contain the disease but continued to deliver care to patients despite feeling vulnerable about their personal safety [42].

Accountability is the core theme that represents the nurses’ experiences of caring for patients with COVID-19. Professional accountability defines the nurse’s legal, ethical, and professional responsibilities. It is based on the obligation of nurses to promote the welfare and wellbeing of the patient through comprehensive and effective nursing practice [43,44]. Professional accountability in nursing is a complex phenomenon that can be compromised by practical or political factors [43,44]. Enhancing professional nursing accountability for patient care during the COVID-19 outbreak requires adequate support from clinical leadership, hospital administration, and the government. Analysis of the data in this study showed that using online resources to obtain new knowledge and strict implementation of infection control protocols enabled nurses to discharge their responsibilities successfully and provide a safe care environment. They accepted professional accountability despite being subject to multiple stresses, including the need to participate in difficult decision making regarding ethical care. Their experience prompted them to rethink the meaning of the care that they provided to patients with COVID-19, which in turn influenced the quality and safety of care and the associated decision-making processes.

One of the limitations of phenomenographic research is the generalizability of research outcomes. This study recruited thirty-four participants, including two male and thirty-two female nurses from three units of two medical centers in Taiwan. The present study did not intend to generalize the research outcomes to a larger population but only to represent Taiwanese nurses’ experiences of caring for patients with COVID-19 during the pandemic.

## 5. Conclusions

The COVID-19 pandemic has increased the stress on nurses and impacted their emotional well-being. The results of this study show that nurses in Taiwan are under enormous stress while caring for patients with this infectious disease and have been negatively affected physically, psychologically, and socially during the pandemic. Nurses’ professional accountability in caring for patients with COVID-19 can be enhanced through adequate support. These findings highlight the responsibility of nursing managers, hospitals, and governments to provide this support by ensuring that sufficient personal protective equipment is available to enable nurses to provide effective and safe patient care. Nursing education should also offer updated knowledge and skills training related to infection control measures in both clinical and community settings. Given the critical work done by nurses and the risks they face during pandemics or outbreaks of infectious diseases, future research should focus on nurses’ resilience following their experience of caring for patients with COVID-19 and the potential role of resilience interventions to improve their well-being. Understanding how nurses build resilience may further enhance professional accountability in nursing.

## Figures and Tables

**Table 1:** Demographics description of participants (*n* = 34).

**Variables**	***n* (%)**	**Mean ± SD**	**Range**
Gender			
Male	2 (5.88)		
Female	32 (94.12)		
Age		30 ± 4.83	22–42
Education			
Associate diploma	6 (17.65)		
Bachelor’s degree	28 (82.35)		
Department (site of recruitment)			
Medical	18 (52.94)		
ICU	8 (23.53)		
ER	8 (23.53)		
Nursing experience (years)		7.61 ± 4.60	1–21

## Data Availability

All data have been illustrated in the manuscript.

## Appendix A. The Interview Guide

1. What was work like today?
2. Could you describe your working situation when the COVID-19 pandemic started?
3. Did you care for any patients with COVID-19 today?
4. Could you provide some examples of how you care for patients with COVID-19?
5. Could you talk about your feelings or thoughts while caring for the patients with COVID-19?
6. Have you had any difficulty while caring for the patients with COVID-19?
7. Do you have any suggestions for caring for the patients with COVID-19?
8. Is there anything you would like to add?
